# To Whom Belongs the Honor of Discovery?

**Published:** 1893-12

**Authors:** 


### Editorial.

### TO WHOM BELONGS THE HONOR OF DISCOVERY?

THE progress of the world is so inextricably made up of atoms of thought, from the period of its earliest manifestations, that to analyze the complex relations of past and present becomes a problem difficult, if not impossible, of solution.

This fact has led many to contemn the idea that there is anything now for the world's workers to present worthy to be classed as novel. The arts lost in the mists of antiquity are an ever-present reminder that the world, in many of its presentations, exhibits an ever-continuing spiral revolution, the fact forgotten yesterday becoming the practical idea of to-day.

While this is unquestionably true, it would be worse than folly to ascribe the development of new things to a combination of the mental effort of former generations. The monuments of by-gone civilizations remind us of periods of growth and decay, but they at the same time show us with ever-increasing force that, while there has been a loss in some directions, the tendency of the world is towards a higher life and grander conception of art, morals, and material things. Were this not the case we might well despair, and adopt the pessimistic idea that progress is a myth, and that the end of the human race is eventually to be, as at the beginning, a lapse into barbarism.

It is to be feared that this view has led many to the unwise conclusion, that there is no such thing as original thought, and, even when this appears as such, it is merely a combination of previous ideas, which some one, more lucky than his fellows, has been able to present in a pleasant and useful form. With this as the governing idea it will be found impossible to convince a certain class of minds that the seeming original conception has any inherent right to be so considered or that the claimant to ownership is entitled to any honor for the successful issue of his labors.

Hence originates the laxity of morals that conceives it no crime to appropriate the ideas of another, and, while the same individual might hesitate to pick his neighbor's pocket or burglariously enter his house at night, he will unblushingly pilfer the coinage of his brain, and feel he has done nothing unworthy an honest man.

The nice ethical distinction often drawn between corporate, political, and professional conscience and the moral standing deemed necessary for Christian people to adopt in their intercourse with each other, is worthy of more consideration than it receives. It is unnecessary to enlarge upon this, but the want of principle involved in this code has become a baneful poison, insidious and far-reaching in its effects..

The question who may claim the honor of original discovery will ever remain a difficult one to answer, if this is meant to apply to one who has shown a capability of formulating an idea without the aid of the work of generations preceding him. To confine this within such narrow bounds would simply be placing an impassable barrier to all progress. The absurdity of this idea needs no comment.

Every investigator must make use of the machinery at his command as aids in development, but the materializing of his conceptions may nevertheless be above and beyond the thought and work of any preceding age.

It is certainly an error to suppose that a fractional idea constitutes an entire conception, yet this would seem to be the thought of many, for the moment a new invention or process is announced there arise numerous claimants for the honor of original discovery. When these claims are subjected to examination, they ordinarily prove to have been ideas in an inchoate condition and incapable of resulting in any form of value.

In illustration of this view it is only necessary to refer to several prominent instances. All are familiar with the history of the discovery of anæsthesia, by Horace Wells, and how the attempts to deprive him of this honor cost him his mental balance and his life. Yet Horace Wells had nothing to do with the discovery of the agent used. Keen observation and quick perception, rather than profound thought, led to the result, but it was just these qualities which enabled him to reason from cause to effect in advance of all others who had made use of nitrous oxide.

It is well known that, when Dr. Bonwill introduced the electric mallet, determined efforts were made to prove that others had preceded him, but, fortunately for justice, these attempts failed; yet Dr. Bonwill was indebted for much of his work, in fact could not have made the mallet, but for the results previously accomplished by electricians,—indeed, the click of the telegraph sounder suggested the idea to him.

Electricians failed to make use of their own discoveries in the direction of sending messages, and it was reserved for Morse to point out the way and be received with deserved honor as the discoverer of the process of telegraphy.

The investigation into the origin of dental caries made slow progress, notwithstanding the work of histologists from Leeuwenhoek to Ficinus, Tomes and Klencke to Leber and Rottenstein, Milles and Underwood, and to the final work of Miller. The foundations were laid before the latter began his labors, but on these he builded better than those who had preceded him, and so thoroughly proved his work as he progressed, that the last word has been said upon the subject or its origin.

When Stebbins proved that nitrate of silver would stop the progress of caries, and demonstrated it by a series of exact and long-continued experiments in the mouth, there arose immediately claimants for the honor of this discovery from all points of the compass; but it was the old story over again, of imperfect experimentation, and missing the vital and important point as demonstrated in deciduous teeth by the real discoverer.

All who have done any labor in a given direction deserve and should receive due credit, but the man who proves his work or makes a practical machine, develops an idea, creates a new ideal in art, or opens up ways which will lead directly to uplifting the burdens of the human family, is a discoverer in the truest and best sense, a blessing to humanity and an honor to his age.

